# Employing learning health system principles to advance research on severe neonatal and paediatric illness in Kenya

**DOI:** 10.1136/bmjgh-2021-005300

**Published:** 2021-03-23

**Authors:** Mike English, Grace Irimu, Samuel Akech, Jalemba Aluvaala, Morris Ogero, Lynda Isaaka, Lucas Malla, Timothy Tuti, David Gathara, Jacquie Oliwa, Ambrose Agweyu

**Affiliations:** 1 Health Services Unit, KEMRI-Wellcome Trust Research Programme Nairobi, Nairobi, Kenya; 2 Oxford Centre for Global Health Research, Nuffield Department of Clinical Medicine, Oxford, UK; 3 Department of Paediatrics and Child Health, University of Nairobi, Nairobi, Kenya; 4 Epidemiology and Demography, KEMRI-Wellcome Trust Research Programme Nairobi, Nairobi, Kenya

**Keywords:** child health, health services research, health systems evaluation, paediatrics

## Abstract

We have worked to develop a Clinical Information Network (CIN) in Kenya as an early form of learning health systems (LHS) focused on paediatric and neonatal care that now spans 22 hospitals. CIN’s aim was to examine important outcomes of hospitalisation at scale, identify and ultimately solve practical problems of service delivery, drive improvements in quality and test interventions. By including multiple routine settings in research, we aimed to promote generalisability of findings and demonstrate potential efficiencies derived from LHS. We illustrate the nature and range of research CIN has supported over the past 7 years as a form of LHS. Clinically, this has largely focused on common, serious paediatric illnesses such as pneumonia, malaria and diarrhoea with dehydration with recent extensions to neonatal illnesses. CIN also enables examination of the quality of care, for example that provided to children with severe malnutrition and the challenges encountered in routine settings in adopting simple technologies (pulse oximetry) and more advanced diagnostics (eg, Xpert MTB/RIF). Although regular feedback to hospitals has been associated with some improvements in quality data continue to highlight system challenges that undermine provision of basic, quality care (eg, poor access to blood glucose testing and routine microbiology). These challenges include those associated with increased mortality risk (eg, delays in blood transfusion). Using the same data the CIN platform has enabled conduct of randomised trials and supports malaria vaccine and most recently COVID-19 surveillance. Employing LHS principles has meant engaging front-line workers, clinical managers and national stakeholders throughout. Our experience suggests LHS can be developed in low and middle-income countries that efficiently enable contextually appropriate research and contribute to strengthening of health services and research systems.

**Summary box**

- Principles informing development of learning health systems include engagement with stakeholders at multiple levels of the health system, generating health information as part of routine work that can be used for quality improvement, service evaluation, surveillance and research, and shared, continuous learning.
- We operationalised these principles to create a ‘horizontal’ Clinical Information Network that has since 2013 engaged with 22 Kenyan hospitals that offer first-referral care to surrounding communities.
- As part of this approach, hospitals have received three monthly quality of care reports and local clinicians, nurses and health information officers have participated in regular and varied peer to peer meetings and activities that span: improvement and process codesign, evidence-based guideline development, research planning and results sharing, and management and communications skills.
- Observational, implementation and interventional research including a cluster and individually randomised controlled trial has or is being conducted using the shared data platform and benefit from access to data typically on thousands of patients meeting inclusion criteria from amongst a growing set of inpatient events now numbering over 250 000.
- Service delivery and health system challenges identified have prompted wider multidisciplinary research, for example, on electronic health records, technology adoption, quality of nursing care and health workers’ roles in leadership, and the strategy has contributed to research capacity development and policy engagement.
- Forms of learning health system can be adapted for and developed in low-income and middle-income countries to enable locally driven research that is directed at the problems seen in routine settings and conducted in partnership with those working in these settings.

## Introduction

In a report published 5 years ago, we argued that learning health systems (LHS) that focus on important outcomes, identify and ultimately solve practical problems of service delivery, drive improvements in quality and test implementation interventions should be formed within low and middle-income countries (LMIC).^1^ We further argued they should involve the full range of patients encountered in routine practice in contrast to more highly resourced and well controlled clinical research facilities. Nonetheless, they should generate new evidence and support rigorous evaluation of intervention effectiveness.^2^ Our thinking drew on important principles informing development of LHS in high-income settings including: (1) creation of a network of engaged and highly motivated stakeholders; (2) developing information tools that staff can efficiently integrate into their routine work so that the same clinical data are entered only once but used for research, surveillance, quality improvement and wider health system performance monitoring; and (3) shared, continuous learning.^2^

For the research and evaluation community LHS might, therefore, overcome the fragmentation and inefficiency resulting from the separation of epidemiological, implementation and interventional research and efforts at quality improvement. At the same time, by operating at broader scale in routine settings they might speed up the research process and promote generalisability of findings.^1^ Their focus on engagement with a wide set of stakeholders, from government to practicing health professionals, patients and communities, might also make them an important framework for evolving embedded research.^3^

Here we aim to illustrate how our paediatric and neonatal research has benefited from efforts to develop an early form of LHS in Kenya. Work to engage stakeholders is described elsewhere.^4 5^ Here, we focus on its contribution to research that involves large and diverse inpatient populations outside tertiary hospital settings. We proceed to highlight areas researchers are being prompted to tackle based on the system challenges identified. We briefly show how the LHS architecture lends itself to initiating new work, including adaptation to conduct surveillance during the COVID-19 pandemic. We conclude with some brief reflections on the value LHS might have in supporting paediatric and neonatal research within LMIC.

## Establishing the Kenyan Clinical Information Network

**Figure 1:**
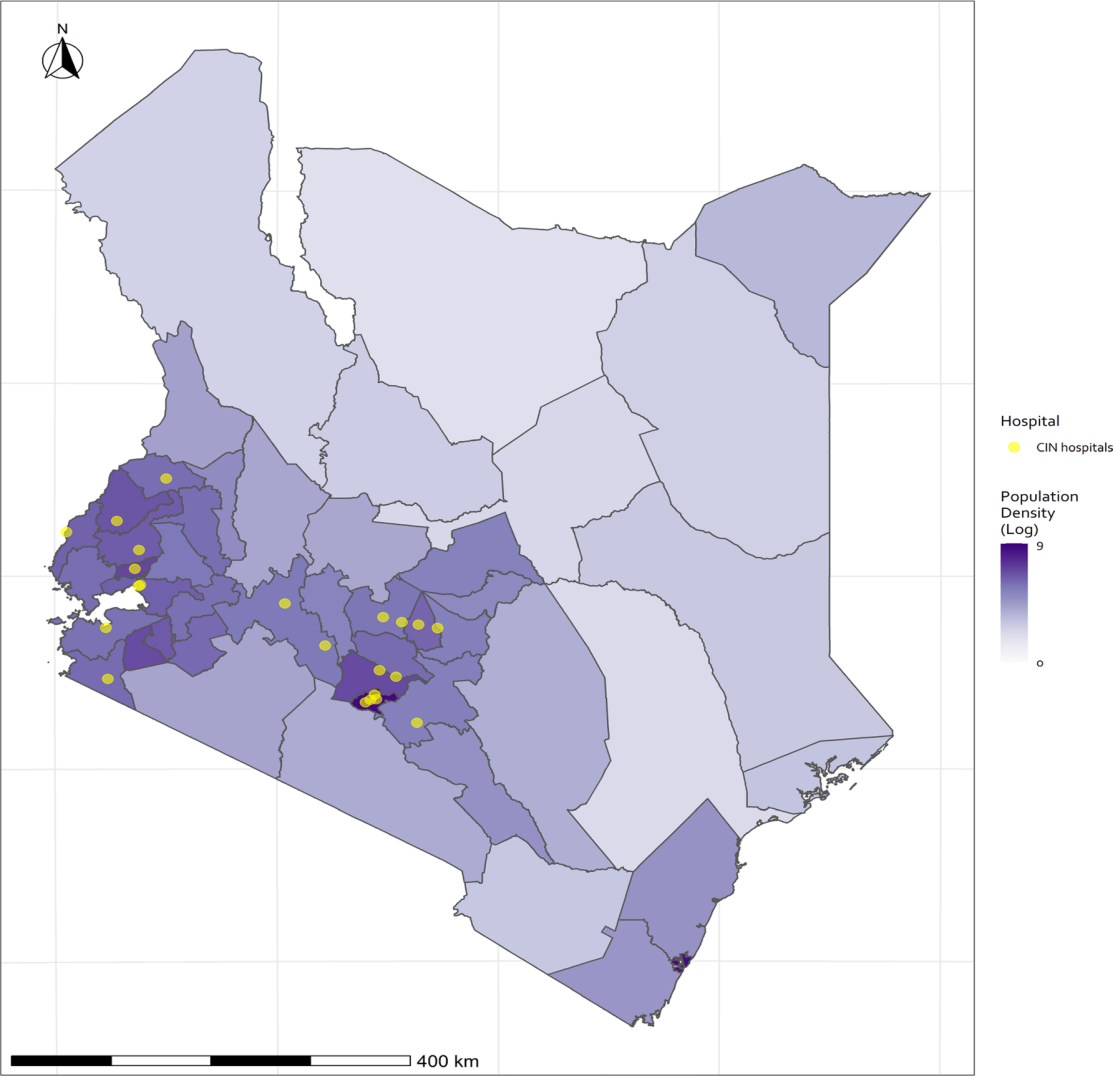
Map of Kenya showing its 47 counties, with darker shading indicating higher population density, and distribution of county hospitals that form the Clinical Information Network (CIN). Hospitals in CIN are not representative of Kenya’s diverse geography as they were purposefully selected but malaria is highly endemic in many counties in the west of the country.

We operationalised LHS principles through a ‘horizontal’ approach engaging with multiple Kenyan county hospitals that offer first-referral care to surrounding communities.^5^ More specifically, we began the Clinical Information Network (CIN) spanning their inpatient paediatric services that admit children aged 0–13 years, subsequently expanding to include inpatient newborn units (NBU) that predominantly admit babies born in the facility who become ill in the first days of life.^6^ Establishing CIN drew on prior collaborative research with partners from the Ministry of Health, the Kenya Paediatric Association and the University of Nairobi.^7 8^ The CIN was initiated in late 2013, 13 purposefully selected county hospitals joined the network by February 2014 (characterised elsewhere^6^) with 22 hospitals participating by the close of 2020 (figure 1 and table 1). CIN has approval from KEMRI’s national Scientific and Ethical Review Unit to operate as a platform generating and analysing deidentified routine data to complement the national health information system, advance health service evaluation and employ data for improvement activities. Any additional research activity necessitating access to personal data or data not captured as part of routine clinical care requires specific ethical approval.

**Table 1:** Summary of key hospital characteristics and important illness specific research findings

District/county hospitals—the context	
Network hospitals were purposefully selected to include counties in high and low malaria endemic settings with moderate to high workloads of between 1300 and 3500 paediatric ward admissions per year.^6^ Pneumonia, diarrhoea with dehydration, suspected meningitis, severe anaemia and severe malnutrition are major causes of admission with comorbidity common, including with malaria and in some settings clinically diagnosed rickets.^6 57^ Laboratories offer limited diagnostic capacity, malaria smears and HIV rapid testing are typically available but simple bedside glucose or urine testing or access to pulse oximetry are often unavailable with almost no access to reliable culture of blood or cerebrospinal fluid microbiology.^5 6^ More specific work demonstrates good access to printed national paediatric and neonatal guidelines but overcrowding and considerable weaknesses in organisation of and material for infection prevention and control in a number of sites.^58 59^ Although larger county hospitals were selected for the network several had no paediatrician in charge of services for long periods and most had high patient to nurse ratios (10–20 patients per nurse being common).	
Inpatient paediatric care—condition specific insights (*total admissions in the studies outlined ranged from 40 000 to 87 000 from which condition specific populations were 16 162 and 1832 for pneumonia in ages 2–59 m and 5–13 years respectively, 8562 and 7657 in two studies of diarrhoea and dehydration, 5766 and 13 104 in two studies of malaria, and of 622 for a study on shock and 5306 on severe acute malnutrition*)	
Pneumonia^18 57^	Although based on admission data only, analyses of data from 17 000 children with pneumonia suggested lower-chest wall indrawing is likely to be a risk factors for poor outcome. Findings prompted policy discussions in Kenya with local adaptation of WHO guidance and ongoing discussion around WHO policy.^26^Those aged 5–13 years represent 20% of all medical admissions with pneumonia responsible for 12% admissions of these and with mortality 8%, there are no evidence-based guidelines for this population.
Malaria^21 22^	In 5 hospitals with large numbers of clinically diagnosed malaria admissions 62% had a confirmatory test result but 69% with a negative result were prescribed antimalarials, most of whom met criteria for severe illness, and only 3.5% cases with an initial negative test had a repeat test as guidelines recommend. In 4 hospitals malaria admissions were further characterised in preparation for evaluating introduction of the malaria vaccine, 40.6% children had severe malaria with a case fatality rate 7.0% and median age 33 months.
Diarrhoea and dehydration^19 20^	Diarrhoea and dehydration remain common causes of admission. Severe illness has a mortality rate of 9%. However, while signs used to diagnose dehydration help guide fluid therapy mortality is more strongly linked to age <12 m and additional signs of severe illness including abnormal circulatory, respiratory or neurological signs. Analyses suggest that mortality is lower in cases where fluids are correctly prescribed and that feedback is associated with documentary evidence of better case management.
Severe acute malnutrition^23^	Severe Acute Malnutrition (SAM) is important in all settings with prevalence and outcomes amongst admissions aged 1–59 m varying from 4.6% to 18.2% and 6% to 28.6%, respectively. Feedback seems to promote use of key anthropometric assessments in diagnosis and while indicators suggest adherence to 5 of the 10 recommended steps in case management is often quite poor there was some evidence it was associated with in small to modest improvements in case management.
Shock^60^	Trials indicated unexpected harm from iv fluid bolus in children with severe febrile illness but no dehydration. Amongst admissions with an overall mortality of 5% fluid bolus was rarely used (0.85%) and 89% boluses given were for severe dehydration; data suggest harm from overuse of boluses is likely to be uncommon where guidelines are clear.

As the initial focus was on paediatric admissions three key focal persons in each hospital were identified. These included the (usually solitary) paediatrician or non-specialist doctor responsible for medical care, the lead nurse on the paediatric ward and the chief health records and information officer.^5^ These hospital staff and other stakeholder groups were invited to once or twice yearly CIN workshops. Workshops were used to discuss individual hospital’s data and that from all hospitals, ongoing improvement activities, new evidence relevant to their practice, and new improvement or research activities.

Given our aim to generate data from routine clinical activity without increasing clinicians’ work, CIN initially improved hospitals’ paper medical records incorporating codesigned checklists to standardise and improve the quality of admission assessments, care planning and discharge.^9 10^ The only material contribution of the CIN to each hospital was a data clerk, a computer and a modem. These allowed data capture from records on patient discharge and secure, deidentified data transmission to a central database. (Data capture, quality assurance and use of 3 monthly reports on quality of care indices are described elsewhere.^10 11^) This paper-to-digital system has been incrementally refined since 2014. Many LHS in high-income countries leverage fully electronic medical records, but research by our team in Kenya highlights the immense challenges posed by transitioning to electronic records at scale while paper-to-digital approaches remain flexible, low-cost and technologically simple.^12^

## Research on severe illness and case management

**Figure 2:**
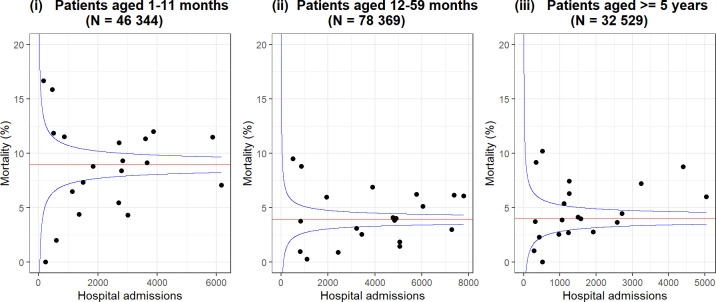
Funnel plots using data from 2013 to 2020 (excluding 2017) from 20 hospitals’ paediatric wards, 2 hospitals that only provide neonatal data are excluded. The plot shows the median mortality proportion (red horizontal line) among paediatric admissions for the infant (1–11 m), child (12–59 m) and older child age groups (≥5 years to a maximum of 13 years). Each hospital’s mortality proportion is indicated by a black circular marker. Variability in size of population available from each site is indicated by their distribution on the X-axis, and variability in mortality proportions across sites indicated by their spread on the Y-axis. Blue lines represent the expected 95% range of hospitals’ mortality proportions assuming their values would be randomly distributed around median mortality.

Despite improvements in under 5 mortality, a small number of conditions account for most deaths and hospital admissions in children in LMIC. WHO provides case management guidance spanning conditions for those aged under 5 through its Pocketbook of Hospital Care for Children.^13^ This broad guidance has been adapted, widely disseminated and updated in the form of Basic Paediatric Protocols in Kenya.^14 15^ Their use is reinforced by complementary training now led by the national paediatric association (this course, ETAT+ and its deployment is described elsewhere^16 17^). The protocols help define common conditions and their severity based on clinical features (eg, in pneumonia) and represent locally agreed, minimal standards of care. Good clinical data allow us to identify patient populations meeting criteria for these conditions and individuals’ severity of illness. CIN data were initially used to better characterise outcomes, risk factors and quality of care for pneumonia, malaria, diarrhoea with dehydration, and severe malnutrition (table 1).^6 18–23^ Findings emphasise that many deaths occur within 48 hours of admission, that mortality proportions vary greatly across hospitals (figure 2), that comorbidity is common and that a set of clinically identifiable risk factors are associated with mortality whatever the clinician’s primary diagnosis. Especially important among these appear to be admission during infancy (age 1–11 m), presence of pallor suggesting underlying anaemia and low weight-for-age without severe malnutrition.^18 19 21^ High and varying prevalence of such risk factors may explain high but varying mortality respectively and is being further investigated (figure 2).^24^

In the particular case of pneumonia findings reignited debate (that continues^25^) around 2013 WHO guidance on the safety of treating children in Africa presenting with lower chest-wall indrawing as outpatients.^18 26^ Findings resulted in a nuanced adaptation of pneumonia guidelines in Kenya recommending outpatient treatment for pneumonia with indrawing only if the family could quickly access a health facility in case of deterioration. Pneumonia studies also highlight it is an important problem in older children (ages 5–13) for whom there are no clinical guidelines.^27^

Encouragingly the data also suggested in some cases that better adherence to guideline recommendations is associated with improved outcomes,^19^ a specific example of a broader association previously observed.^28^ Sadly, the data also provide quantitative evidence of low adherence to minimal best practice recommendations often linked to inability to provide reliable access to even basic interventions or diagnostic tests.^5 6^ Thus, in the management of severe acute malnutrition therapeutic feeding seems poorly planned and provided and hospitals seem unable to offer children with severe illness reliable access to blood glucose or bedside urine testing.^6 23^ Meanwhile, availability of blood or spinal fluid cultures is almost universally absent undermining our ability to understand and respond to the threat of antimicrobial resistance.^6^ Indeed lack of access to diagnostics with the exception of malaria, haemoglobin, and HIV testing means most treatment decisions are based only on clinical findings as we enter 2021.

## Implementation learning

In keeping with the principles of LHS, the CIN used the common data to improve quality by generating routine feedback reports focusing on performance in adhering to consensus-based guideline recommendations drawing on earlier intervention studies.^29 30^ This is the first regular feedback of its type to be provided on inpatient care in Kenya at scale. Feedback was combined with outreach/mentorship, peer networking and some team leader training.^5^ The rationale for these strategies is discussed fully elsewhere.^4 31^ Performance improvements were observed across hospitals and for many indicators.^5 20 30^ However, improvements often took many months and varied across hospitals. Local leadership seemed key to sustained success especially in establishing new routines in settings where the junior clinical staff change every few months, while difficulties implementing some minimal practice standards were hampered by inability or hesitancy of senior managers to act, especially when resource mobilisation was required (eg, availability of glucose testing or pulse oximeters).^32 33^ In this regard, implementation challenges likely reflect deep-seated financing, governance and priority setting challenges.^34^

Having established routine feedback systems we were able to progress our implementation research by conducting a cluster randomised trial. This suggested that enhancements to feedback might promote more rapid adoption of a new antibiotic policy.^35^ In doing this, we demonstrated that LHS can be a platform for experimental designs tackling globally relevant questions on feedback systems.^36^

The CIN also provided opportunities to explore specific implementation issues in depth using mixed methods. Access to and use of pulse oximetry in Kenyan hospitals were very limited when CIN began. Employing only feedback on use of pulse oximetry we did observe some improvements in use of this basic, cheap technology but in only half of CIN hospitals.^37^ Detailed qualitative research revealed that failures in procurement and maintenance of pulse oximeters were key reasons for implementation failure compounded by inadequate local supervision and training of frontline staff.^37^ In the case of Tuberculosis (TB) access to Xpert MTB/RIF, the purchasing challenge was overcome through centralised supply of technologies to hospitals by government. However, data only available from CIN showed utilisation of both this technology and traditional diagnostics to diagnose paediatric TB was very poor.^38^ Here, qualitative research suggested the national programme’s omission to engage relevant hospital leaders, poorly structured patient pathways and stigma, together with inadequate skills building of frontline staff, all contributed to implementation failure, understanding that can inform future interventions.^39^

## Health system challenges

The CIN highlights clinical consequences of broader health system weaknesses. Admission with severe anaemia is a relatively common, life-threatening event where malaria is highly endemic.^6 21^ In a study of over 1000 emergency transfusions for anaemic children 25% could not be given on the day they were ordered. In this group mortality rose to 20% compared with 12% among those transfused on the same day.^40^ Here, weaknesses spanning the clinical, laboratory and national blood transfusion services all conspire to increase the risk of mortality.

CIN data were for the first time also able to demonstrate how reliant many hospitals are on prelicensure clinician interns who are responsible for the assessment and management of over 80% of paediatric ward admissions.^41^ These inexperienced staff seem to struggle in correctly assigning the severity of illness resulting in overprescription of some treatments.^41^ Another critical workforce concern is illustrated by findings that nursing staff can rarely manage regular patient monitoring, even for the most severely ill. Furthermore, when such monitoring is performed, the observations may be unreliable.^42^

The important impacts of inadequacies in workforce numbers and skills on quality and implementation of new practices and technologies have prompted wider research linked to the CIN on local leaders’ roles and the consequences of high patient to nurse ratios.^33 43^ Little attention seems paid to the financing of Kenya’s ambitious strategy to close workforce gaps.^44^ As a result, it is likely that all innovations and technologies aiming to improve facility-based care will suffer the implementation challenges we highlight.^5 37 38^ Challenges with health system financing and governance, although not subjects we tackled directly, are perhaps most clearly demonstrated using CIN data showing an 80% fall in paediatric and neonatal admissions in 2017 as a result of prolonged health worker strikes.^45^

## Continuing development and adaptation of CIN

The CIN as an early form of LHS began by engaging 13 hospitals. One, the smallest, could not sustain participation of focal persons. However, the networking approach and growing local interest have enabled new programmes to start that together have increased participation to a total of 22 hospitals by the close of 2020. New programmes include employing the CIN to track the impact on severe disease of the malaria vaccine, conduct of a large, individually randomised controlled trial of severe pneumonia^46^ and support to help sustain essential services through the COVID-19 pandemic. Building on development of neonatal data systems in one facility an expansion to include NBU, largely in facilities already contributing paediatric data, began in 2018.^47^ This neonatal expansion is also supported by several initiatives including the multicountry NEST360 programme that is introducing needed technologies and aiming to demonstrate improvement in quality and outcomes.^48^ As a result by December 2020, data on approximately 180 000 paediatric and 70 000 NBU admissions have been collected and used to provide feedback. These data will provide important insights into long-term changes in disease and outcomes.^49^

All of these initiatives have involved partnership with government, other national organisations and hospitals. As the COVID-19 pandemic evolved one consequence was specific extension of CIN to rapidly initiate surveillance of adult medical ward admissions.^50^ Data are being used to identify what proportion of paediatric and adult admissions have severe acute respiratory illness, track whether SARS-COV-2 infection is confirmed, and give rapid feedback to the government.^50^ More broadly as data collection has largely been sustained throughout 2020, they will provide future insights into the impact of COVID-19 on utilisation of Kenyan hospitals.

## Lessons learnt

We believe our emphasis on deploying paper-to-digital information tools so that clinicians continue with familiar work routines, rather than trying to leapfrog and introduce electronic records, has helped achieve success at relatively low cost. However, all data systems need constant attention to optimise and sustain data quality, to allow for inevitable revisions and updates and to service data and analysis requests. LMIC invest relatively little in data systems and the critical skilled human resources they require. The CIN team has invested in building a core and crucially important skilled team including clinical epidemiologists, statisticians/data managers and administrative staff. Hospitals are supported with only one or in especially busy locations two data entry staff. The cost of the first 4 years of operations and linked research was approximately £3 million, including all the most senior staff. Even basic LHS elsewhere will require similar investments. This raises questions of their sustainability in LMIC that lack the billions of dollars invested in health information in high-income countries.^51^ However, this cost is similar to that often required to run a single, large multicentre randomised controlled trial and it is not uncommon for governments and partners to invest similar sums annually on short in-service training courses such as those for malaria. Furthermore, there are economies of scale as the incremental costs of adding new sites can be relatively low once a central expert team is established and such platforms can attract new funding as we have seen.

Establishing LHS is not without challenges. Despite paying considerable attention to promoting quality all routine data have limitations. The data collected are documented by frontline clinicians with varying levels of skill and often under time pressure. The primary data may not be an accurate representation of a patients’ actual condition.^52^ Data also only capture events at a few specific timepoints, thus painting a very limited picture of an entire admission, and we must assume that what is documented is what is done. These and other factors make data in paper-to-digital systems or electronic records prone to errors and biases. In paper-to-digital systems further transcription errors may be introduced. CIN makes efforts to limit all these challenges but cannot eliminate them.^10 11^ Problems also arise when some data are missing. Many clinical reports are based on complete cases assuming data are missing completely at random. We have increasingly employed methods for multiple imputation that assume data are missing at random to maximise data use, but these and those based on complete cases may be biased where data are missing not at random.^53 54^ For these reasons and others, and because hospitals were purposefully included all specific findings must be interpreted cautiously.

After 7 years spanning a global pandemic, our early efforts to form a LHS focused on paediatric and neonatal admissions to first referral hospitals in Kenya have been rewarded by development of an extensive network of national and local stakeholders who have all contributed to successes (see CIN author group list). This fulfils in part one of the other guiding LHS principles.^1 2^ Many improvements can be made now to quality of care in multiple locations that may influence inpatient outcomes, not all of them initially anticipated.^55^ However, our work and experience also illustrate how difficult it is in LMIC to address system weaknesses that span the apparently basic, such as access to blood glucose testing, to the profound, such as workforce deficits.^5 43 44^ In the future better means to use the data generated to support prioritisation of investments, management and accountability are needed.

The CIN has resulted in wider benefits to the research system in Kenya. It has helped develop the relatively neglected field of health services research and local implementation science capacity. Over 50 CIN-linked international peer-reviewed publications by Kenyan first or senior authors have been produced, and 6 Kenyan scientists have completed PhDs with a further 4 in progress. We also continue to reflect on how to employ theory to produce change in hospitals at scale to promote generalisable learning.^4 31 39 56^

## Conclusion

In conclusion, we believe that low-cost LHS can be an important addition to the paediatric and neonatal research landscape in LMIC. We hope that research funders and other partners begin to appreciate their potential to address a broad range of concerns, their relative efficiency, potential for responsive research and policy impact, and advantage in promoting cumulative learning. While improvements can always be made we believe there are strong grounds to promote investment in such systems to widen geographic coverage and extend to other clinical arenas including outpatients. In doing this they should work synergistically to strengthen national information systems to provide detailed data in the short term and model future disease surveillance, technology adoption and performance monitoring strategies. Such platforms may also support programme evaluation and conduct of large pragmatic clinical trials. We should not forget however that CIN, and LHS in general, are primarily comprised individuals and organisations working in respectful partnerships and that investment in these human elements remains critical to their success.
